# Aposematism facilitates the diversification of parental care strategies in poison frogs

**DOI:** 10.1038/s41598-021-97206-6

**Published:** 2021-09-24

**Authors:** Juan D. Carvajal-Castro, Fernando Vargas-Salinas, Santiago Casas-Cardona, Bibiana Rojas, Juan C. Santos

**Affiliations:** 1 Department of Biological Sciences, St. John’s University, Jamaica-Queens, NY USA; 2 Grupo de Evolución, Ecología y Conservación (EECO), Programa de Biología, Universidad del Quindío, Armenia, Colombia; 3 Department of Biology and Environmental Science, University of Jyväskylä, Jyväskylä, Finland; 4 Department of Interdisciplinary Life Sciences, Konrad Lorenz Institute of Ethology, University of Veterinary Medicine Vienna, Vienna, Austria

**Keywords:** Behavioural ecology, Evolutionary ecology, Phylogenetics

## Abstract

Many organisms have evolved adaptations to increase the odds of survival of their offspring. Parental care has evolved several times in animals including ectotherms. In amphibians, ~ 10% of species exhibit parental care. Among these, poison frogs (Dendrobatidae) are well-known for their extensive care, which includes egg guarding, larval transport, and specialized tadpole provisioning with trophic eggs. At least one third of dendrobatids displaying aposematism by exhibiting warning coloration that informs potential predators about the presence of defensive skin toxins. Aposematism has a central role in poison frog diversification, including diet specialization, and visual and acoustic communication; and it is thought to have impacted their reproductive biology as well. We tested the latter association using multivariate phylogenetic methods at the family level. Our results show complex relationships between aposematism and certain aspects of the reproductive biology in dendrobatids. In particular, aposematic species tend to use more specialized tadpole-deposition sites, such as phytotelmata, and ferry fewer tadpoles than non-aposematic species. We propose that aposematism may have facilitated the diversification of microhabitat use in dendrobatids in the context of reproduction. Furthermore, the use of resource-limited tadpole-deposition environments may have evolved in tandem with an optimal reproductive strategy characterized by few offspring, biparental care, and female provisioning of food in the form of unfertilized eggs. We also found that in phytotelm-breeders, the rate of transition from cryptic to aposematic phenotype is 17 to 19 times higher than vice versa. Therefore, we infer that the aposematism in dendrobatids might serve as an umbrella trait for the evolution and maintenance of their complex offspring-caring activities.

## Introduction

Many organisms evolve morphological or behavioral adaptations that increase offspring survival^1,2^. In animals, these strategies commonly include elaborate nest building^3,4^, costly periodic food provisioning^5,6^ and aggressive defense of their progeny^7^. These adaptions have evolved independently several times across vertebrates, and have been shown to increase the likelihood of offspring reaching reproductive age. However, these parental care strategies often entail significant life history trade-offs for parents, such as the reduction of their reproductive output while caring for their offspring^8–12^.

Parental care investments fall within a continuum that ranges from limited or no protection to prolonged offspring attendance. In one extreme of this spectrum, species that present limited or no parental care usually have hundreds of offspring, few of which survive after birth and only a fraction of which reach maturity^13^. Many such low-parental care taxa have repeated bouts of reproduction, and their offspring are relatively inexpensive to produce (i.e., small seeds, tiny larvae or thousands of eggs^14^). On the opposite side of the parental care spectrum, species with elaborate and prolonged care of their young (e.g., brood attendance and provisioning) aim their care at a small number of high-quality offspring whose survival is significantly increased by a consistent input of food (energy) and protection during early development. For parents, such behaviors are costly in terms of self-maintenance and the loss of future reproductive events^1,2,13,15^.

Understanding the parental care spectrum and its associated trade-offs requires dissecting a syndrome of behaviors into their individual components (e.g., ethological and morphological correlates). These components must be integrated into models that reveal the underlying parental care architecture in specific species as emergent properties. Likewise, other aspects of the natural history of focal species (e.g., anti-predator strategies) might affect the trade-offs that parental care can impose. Finding such cost–benefit relationships is a significant endeavor in evolutionary ecology, especially when trying to determine how individuals balance offspring-rearing and self-maintenance^16^.

To best elucidate the evolutionary trajectories leading to parental care, we must characterize how diverse morphological traits (e.g., body size) or antipredator strategies (e.g., aposematism or crypsis) correlate with parental care components (e.g., number of offspring, food provisioning, and male-/female-biased attendance). To study such complexity, we must focus on a group of closely related species with diverse phenotypes regarding reproduction and parental care, and account for interspecific variation in life history traits including sexual size dimorphism (male-biased vs. female-biased), number and quality of offspring (i.e., few vs. many), and asymmetric parental care investment (i.e., absence vs. extensive biparental care). In addition, we should focus on clades with innate (i.e., not learned) forms of parental care, which would reveal how natural selection affects parental care evolution without the confounding effects of learning. Finally, an ideal study system should involve a lineage with recurrent and contrasting types of parental care to allow the effective use of phylogenetic comparative methods.

Among vertebrates, parental care is common in birds and mammals^17–19^, and present in some reptiles and fishes^20–22^. In amphibians, ~ 90% of species do not display parental care, but the remaining species exhibit a wide diversity of caring behaviors that are innate and seemingly shaped by natural selection^23,24^. Neotropical poison frogs (i.e., Dendrobatidae) form a clade that evolved from other hyloid frogs at ~ 40 MYA, and many species have diverse forms of parental care. Such behavioral variability has stimulated extensive research on parental care origins, maintenance, and trade-offs among anurans, which dates back to the 60’s^24–26^. Parental care diversity in dendrobatids ranges from a few taxa without any form of offspring care to species with male only or male-biased care (comprising the majority of species), and some with extensive biparental care including the provisioning of unfertilized eggs to developing tadpoles by their mothers^27^. In sum, dendrobatids are characterized by small clutches of terrestrial eggs (mean = 12.41 ± 9.26 eggs, range = 1–40, N = 107 species; see Table S2) which are cared for by one parent, mostly the male, until tadpoles hatch and are transported to a water body^24,27^.

In most dendrobatids, tadpoles are transported to small streams, ponds (hereafter referred to as  non-phytotelma) or bodies of water in plant structures (hereafter referred to as phytotelmata) where development continues until metamorphosis. In some species with large clutches, the transport of all tadpoles requires several trips by the attending parent^26,27^. Pools in phytotelmata (i.e., tree-holes, palm bracts, bromeliad axils, etc.) offer a suitable rearing environment where large predators are rare, but nutritional resources are scarce^28^. Thus, only a few tadpoles (e.g., 1–2 individuals) are transported to, and deposited in, these environments at the same time by a given parent, and it is not uncommon for larval cannibalism to occur. Because of resource scarcity in phytotelmata, some species go one step further and periodically provision their developing tadpoles with unfertilized eggs^29–31^. Both tadpole transport and food-provisioning visits are done during the day and often involve trips over long distances, which may increase the exposure of the caring parent to predators, and presumably incurs significant energetic costs^32–34^.

Alongside their elaborate parental care, another feature common to many dendrobatids is aposematism^35–37^, an antipredator strategy defined as the coupling of a warning signal (e.g., warning coloration) and a secondary (e.g., chemical) defense^38^. In this clade, ~ 100 species exhibit aposematism, while the remaining ~ 210 species^39,40^ are assumed to use crypsis to blend with their environment and avoid predator detection or recognition. The origin of warning coloration in dendrobatids is likely associated with their diurnal habits, while the chemical defenses are likely derived from a specialized diet consisting mostly of ants and mites^39^, followed by the sequestration of toxic alkaloids from these key arthropod prey^41,42^. Furthermore, aposematism has been co-opted to provide an extensive audiovisual communication channel during mating and territorial signaling, and has played a central role in the diversification of poison frogs^43^.

To date, the implications of aposematism for reproductive strategies across the Dendrobatidae (i.e., its ~ 310 species) have not been explored extensively. Previous comparative analyses in this clade suggest that aposematism likely affects most, if not all, aspects of dendrobatid life history^35,39,43–45^. Such analyses have also suggested that male-only care may have preceded and favored the emergence of aposematism^46^, and that tadpole deposition in large bodies of water is likely the ancestral state^27,47^. However, both macroevolutionary (e.g.,^27,47,48^) and empirical^49–57^ studies on reproductive behavior in dendrobatids are limited to a few, predominantly aposematic species, while most non-aposematic species (i.e., ~ 210 species) have been largely overlooked (but see e.g.,^57–61^ for detailed studies on the reproductive biology of cryptic species). In spite of this bias, most observations suggest that aposematic species (e.g. *D. pumilio* and *D. imitator* mimicry system^63^) tend to have more complex reproductive behaviors than cryptic species, involving elaborate audiovisual and tactile interactions (e.g.,^49,54,61,63,64^) in which warning colors might be used as cues by females to select mates^65^. Likewise, many aposematic species have evolved female-biased or female-only care, which involves spatial field-mapping abilities to repeatedly locate their developing tadpoles in phytotelmata and provision them with unfertilized eggs^26,29,47^.

Here, we aim to determine whether aspects of parental care behavior in dendrobatid frogs, such as sex of the main caregiver, clutch size, and the transition from streams and ponds to ephemeral deposition sites (i.e., phytotelmata), may have evolved in association with the aposematic phenotype across the entire family. To do so, we characterized the morphological and behavioral aspects of parental care in dendrobatids using phylogenetic multivariate tools^35^. More specifically, we (1) estimated the ancestral state reconstructions and used multivariate models to determine how aposematism is related to parental care; (2) established whether aposematism and tadpole traits are related using phylogenetic multivariate logistic regressions; and (3) tested the transition rates between aposematism components (i.e., conspicuous coloration and chemical defenses) and phytotelm-breeding behavior. We found no evidence of a direct relationship between aposematism and offspring morphological traits, but found that aposematic species tend to use more specialized tadpole-rearing sites (e.g., phytotelmata), and ferry fewer tadpoles than non-aposematic species. In addition, we found that in phytotelm-breeders the rate of transition to aposematic phenotype is 17–19 times higher than its reversal to the ancestral cryptic, non-defended phenotypes. We thus propose that aposematism served as an “evolutionary platform” where a diversity of behaviors could evolve as predation pressure decreased. While we do not claim that aposematism causes complex parental behaviors to evolve in the first place, we suggest that it might serve as an umbrella trait that provides protection from predators while allowing, in turn, further diversification of parental care behaviors.

## Results

### Ancestral reconstructions

**Figure 1:**
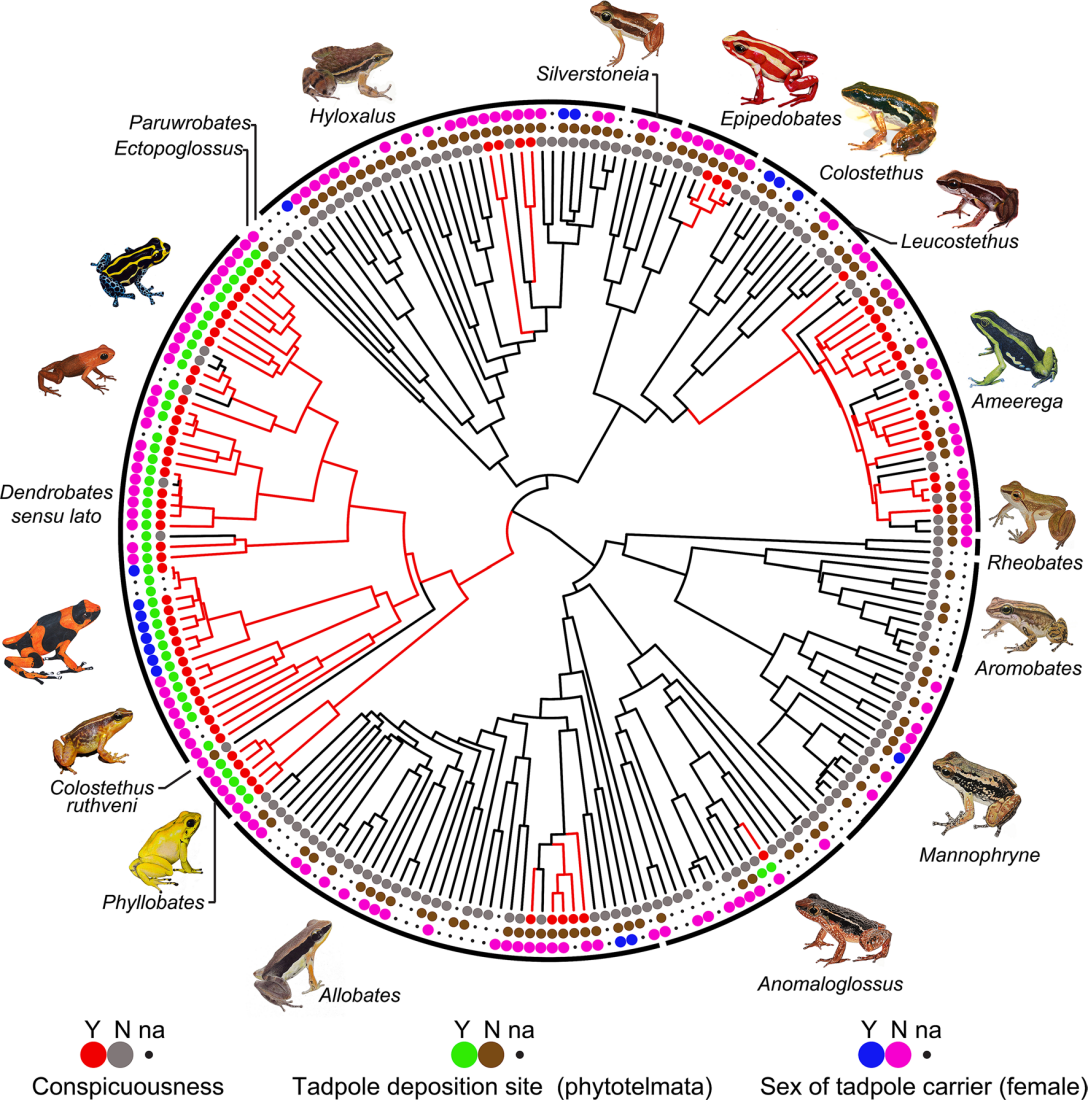
Ancestral reconstruction of coloration, tadpole deposition site and sex of tadpole carrier in Dendrobatidae. Illustrations by MJ Tovar-Gil, AM Ospina-L and DL Rivera-Robles. Figure was created using R software version 4.0.3 (https://www.r-project.org/) and modified using Adobe Illustrator version 15.1.0 (https://www.adobe.com/).

**Figure 2:**
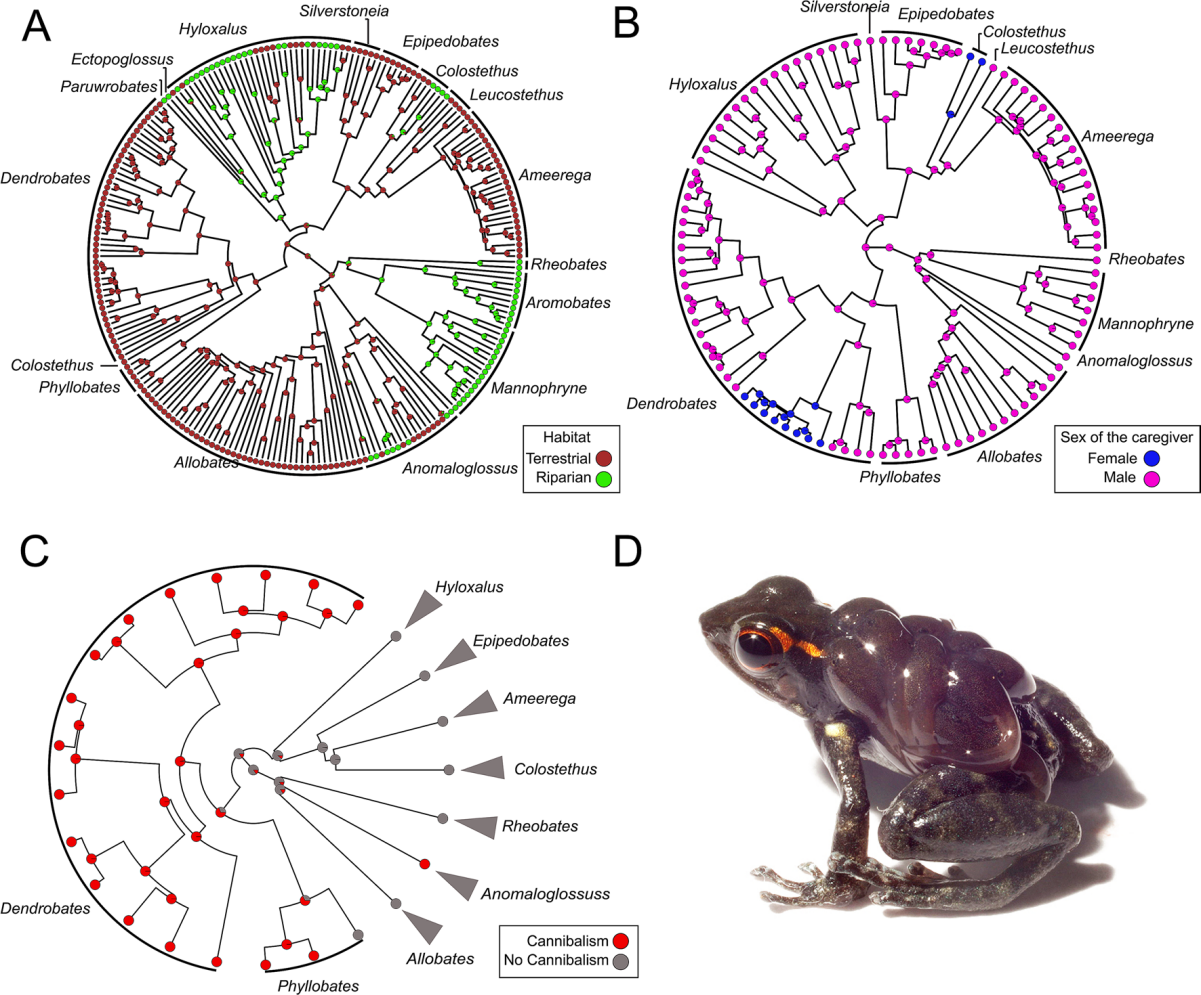
Ancestral reconstruction of (**A**) habitat, (**B**) sex of caregiver and (**C**) larval cannibalism in Dendrobatidae. (**D**) A male *Hyloxalus nexipus* transporting tadpoles. Photograph by Juan C. Santos. Figures were created using R software version 4.0.3 (https://www.r-project.org/) and modified using Adobe Illustrator version 15.1.0 (https://www.adobe.com/).

We obtained information on morphological and behavioral traits of 220 dendrobatid species (Fig. 1, Table S2). We found males to be the caregiver in most cases (92 species), and ancestral reconstructions support this as the ancestral condition in Dendrobatidae; female care (10 species) evolved independently at least two times, once within the genus *Colostethus* and once in the genus *Dendrobates *sensu lato (Tables 1, 2, Fig. 2B). Tadpole transport by males (132 species) is ratified as the ancestral condition in dendrobatids, whereas tadpole transport by females (17 species) has appeared at least five times in the genera *Dendrobates* (subgenus: *Oophaga*), *Allobates*, *Hyloxalus*, *Colostethus,* and *Mannophryne* (Tables 1, 2)*.* Tadpole deposition in non-phytotelmata is more common (112 species) than in phytotelmata (52 species), and non-phytotelm breeding is supported as the ancestral condition. Changes in the tadpole deposition site from non-phytotelmata to phytotelmata have evolved independently two times, once in the aposematic *Dendrobates* (subgenera: *Adelphobates, Andinobates, Dendrobates, Excidobates, Minyobates, Oophaga, Ranitomeya*; 45 species in total), and once in the non-aposematic genus *Anomaloglossu*s (only 3 species; Fig. 1).

**Table 1:** Parental care traits in dendrobatid species considered to be aposematic. The symbol “–” denotes unknown data.

Trait	*Dendrobates sensu lato*									
	*Adelphobates*	*Andinobates*	*Dendrobates*	*Excidobates*	*Minyobates*	*Oophaga*	*Ranitomeya*	*Phyllobates*	*Ameerega*	*Epipedobates*
**Ntaxa**	3	12	4	3	1	10	17	5	29	7
**♂ body size (mm)**	23.727 ± 8.471 (3)	15.617 ± 2.128 (11)	32.525 ± 6.408 (4)	20.695 ± 5.133 (3)	14 (1)	25.639 ± 6.05 (8)	16.09 ± 1.436 (16)	29.54 ± 8.89 (5)	24.55 ± 5.13 (25)	17.75 ± 1.71 (7)
**♀ body size (mm)**	26.16 ± 8.606 (3)	16.117 ± 2.344 (11)	35.975 ± 7.514 (4)	22.103 ± 5.781 (3)	16 (1)	25.759 ± 5.507 (8)	17.454 ± 1.506 (16)	32.48 ± 8.50 (5)	27.46 ± 6.03 (25)	19.05 ± 2.19 (6)
**Habitat**	3	10	4	3	1	9	17	5	27	7
Near streams	–	–	–	–	–	–	–	–	–	–
Far streams	3	10	4	3	1	9	17	5	27	7
**Clutch size**	5.633 ± 3.925 (3)	2.425 ± 1.15 (4)	5.4 ± 1.451 (4)	8 ± (1)	6 (1)	6.43 ± 2.526 (8)	3.009 ± 1.46 (14)	15.8 ± 2.95 (5)	18.43 ± 10.18 (16)	17.75 ± 6.34 (4)
**Neggs**	2.4 ± (1)	3.75 ± 2.475(2)	7 ± 2.309 (4)	–	–	5.333 ± 1.538 (6)	3.464 ± 1.595 (11)	18.87 ± 7.59 (4)	18.53 ± 8.02 (7)	19.37 ± 3.73 (4)
**Egg size (mm)**	–	2.49 ± 0.875 (3)	3.495 ± 0.816 (4)	1.35 ± (1)	–	1.506 ± 0.403 (5)	1.783 ± 0.409 (7)	2.33 ± 0.29 (3)	2.26 ± 0.77 (9)	1.33 ± 0.09 (4)
**Tadpole size (mm)**	14.94 ± (1)	12.586 ± 3.871 (6)	14.096 ± 3.106 (4)	14.987 ± 4.255 (3)	11.796 (1)	10.156 ± 0.791 (6)	14.41 ± 3.032 (13)	11.41 ± 1.16 (4)	12.93 ± 1.95 (15)	10.57 ± 1.49 (6)
**Ntadpoles carried**	1 ± 0 (3)	1.45 ± 0.369 (10)	1.75 ± 0.289 (4)	1.5 ± 0 (2)	2 (1)	1.016 ± 0.044 (8)	1.346 ± 0.718 (13)	5.87 ± 1.88 (5)	11.61 ± 6.75 (19)	8.52 ± 4.41 (7)
**Ntaxa (Cannibalism)**	1	1	4			3	6		1	1
Present	1	1	4		–	3	6	6	–	–
Absent	–	–	–	–	–	–	–	–	1	1
**Caregiver (Ntaxa)**		6	4	2		10	13	5	17	7
♂	–	6	4	2		–	–	5	17	7
♀	–	–	–	–		8	11	–	–	–
Both	–	–	–	–		2	2	–	–	–
None		–	–	–	–	–	–	–	–	–
**Transporter (N taxa)**	3	10	4	2	1	10	14	5	19	7
♂	3	10	4	2	1	–	14	5	17	7
♀	–	–	–	–	–	8	–	–	2	–
Both	–	–	–	–	–	2	–	–	–	–
None	–	–	–	–	–	–	–	–	–	–
**Deposition site (N)**	1	10	4	3	1	10	16	5	–	7
Phytotelmata	1	10	4	3	1	10	16	5	–	–
Non–phytotelmata	–	–	–	–	–	–	–	–	17	7
**Conspicuousness**^a^	2.702 ± 0.219 (3)	2.452 ± 0.293 (11)	2.449 ± 0 (4)	1.966 ± 0.837 (3)	2.646 (1)	2.449 ± 0 (8)	2.525 ± 0.198 (17)	2.53 ± 0.11 (5)	2.51 ± 0.24 (26)	2.51 ± 0.54 (7)
**Alkaloids**^a^	–	6.217 ± 0.978 (2)	7.179 ± 0.234 (3)	–	–	7.152 ± 0.222 (5)	6.909(1)	5.52 ± 0 (3)	6.91 ± 0 (4)	6.91 ± 0 (2)

**Table 2:** Parental care traits in dendrobatid species considered to be non-aposematic. The symbol “–“ corresponds to unknown data. ^a^Conspicuousness and alkaloids scores are described in the methods section; *These species are nidicolous. Vales are reported as mean ± SD (number of data).

Trait	*Al*	*An*	*Ar*	*Co*	*Ec*	*Hy*	*Le*	*Ma*	*Par*	*Rh*	*Si*
**Ntaxa**	75	25	11	9	2	40	10	17	1	3	5
♂ **body size (mm)**	18.94 ± 3.51 (43)	18.45 ± 2.29 (17)	25.54 ± 8.33 (10)	23.81 ± 4.99 (5)	20.33 (1)	21.21 ± 3.68 (34)	20.62 ± 1.60 (5)	24.05 ± 4.77 (17)	21 (1)	31.4 ± 5.3 (2)	16.71 ± 0.84 (4)
♀ **body size (mm)**	19.57 ± 3.32 (42)	20.74 ± 3.43 (15)	28.87 ± 10.19 (10)	25.62 ± 3.68 (6)	23.6 ± 1.41 (2)	23.31 ± 3.99 (34)	22.12 ± 1.89 (6)	27.71 ± 5.22 (17)	23.7 (1)	35.1 (1)	18.67 ± 1.72 (5)
**Habitat**	42	18	10	7	–	33	5	17	1	2	5
Near from Stream	–	8	–	3	–	21	1	17	1	2	–
Far from Stream	42	8(2b)	10	4	–	12	4	–	–	–	5
**Clutch size**	16.63 ± 9.57 (21)	4.59 ± 1.79 (6)	–	25 (1)	–	16.28 ± 7.64 (12)	16.5 ± 3.53 (2)	18.42 ± 10.39 (4)	–	23.5 (1)	–
**Neggs**	16.39 ± 9.82 (13)	4 ± 1.41 (2)	–	–	–	21.01 ± 11.37 (11)	22 ± 4.24 (2)	11.44 ± 1.78 (2)	–	23.5 (1)	–
**Egg size (mm)**	1.69 ± 0.5 (15)	2.6 ± 0.94 (4)	1.5 (1)	1.7 ± 0.14 (2)	2.5 (1)	2.04 ± 0.56 (12)	1.29 (1)	2.21 ± 0.96 (5)	–	3.35 (1)	–
**Tadpole Size (mm)**	11.44 ± 4.32 (23)	16.94 ± 3.49 (7)	–	12.47 ± 0.49 (3)	–	16.06 ± 6.89 (28)	13.6 ± 0 (1)	16.29 ± 3.69 (10)	9.32 (1)	21.2 (1)	11.33 ± 1.04 (3)
**Ntadpoles carried**	11.75 ± 6.68 (24)	3.64 ± 3.54 (7)	–	15.5 ± 9.13 (3)	–	9.45 ± 4.35 (25)	12 ± 2.82 (2)	9.43 ± 3.08 (7)	3 (1)	15.9 (1)	6.67 ± 2.02 (3)
**N taxa (Cannibalism)**	–	–	–	–	–	1	–	–	–	1	–
Present	–	–	–	–	–	–	–	–	–	–	–
Absent	–	–	–	–	–	1	–	–	–	1	–
**Caregiver (N taxa)**	15	4	–	2	–	18	1	7	–	1	1
♂	12	1	–	–	–	17	1	7	–	1	1
♀	–	–	–	2	–	–	–	–	–	–	–
Both	1	2	–	–	–	1	–	–	–	–	–
None	2*	1	–	–	–	–	–	–	–	–	–
**Transporter (N taxa)**	24	10	–	4	–	25	3	8	1	1	3
♂	14	9	–	1	–	20	3	7	1	1	3
♀	2	–	–	3	–	1	–	1	–	–	–
Both	6	–	–	–	–	4	–	–	–	–	–
None	2*	1	–	–	–	–	–	–	–	–	–
**Tadpole Deposition**	27	11	4	3	–	29	4	10	1	1	3
Phytotelmata	2*	3(2*)	–	–	–	–	–	–	–	–	–
Non-Phytotelmata	25	6	4	3	–	29	44	10	1	1	3
Conspicuousness^a^	1.55 ± 0.45 (38)	1.41 ± 0.57 (16)	1.44 ± 0.69 (10)	1.79 ± 0.31 (7)	1.41 (1)	1.59 ± 0.46 (33)	2.09 ± 0.22 (5)	1.87 ± 0.14 (17)	1.41 (1)	1.57 ± 0.22 (2)	1.60 ± 0.17 (5)
Alkaloids^a^	0 ± 0 (7)	–	0 (1)	0.15 ± 0.23 (2)	–	0 ± 0 (3)	0 (1)	–	–	–	0 (1)

Most dendrobatids (164 species) have home ranges that are not adjacent to streams, and this was reconstructed as the ancestral condition for the family; shifts towards habitats near streams (64 species) have occurred eight times in non-aposematic genera including *Anomaloglossus*, *Aromobates*, *Colostethus*, *Ectopoglossus*, *Hyloxalus*, *Leucostethus*, *Mannophryne*, *Paruwrobates*, and *Rheobates* (Table 2, Fig. 2A, D, for an example). Tadpole cannibalism has evolved only twice; in *Anomaloglossus beebei* and in the clade formed by *Phyllobates* and *Dendrobates *sensu lato (subgenera: *Adelphobates, Andinobates, Dendrobates *sensu stricto, *Oophaga* and *Ranitomeya*; 15 species in total; Fig. 2C). Non-conspicuous coloration (143 species) is the ancestral state in dendrobatids (Fig. 1), whereas conspicuous coloration (77 species) evolved independently at least seven times across the family in the genera *Allobates*, *Ameerega*, *Anomaloglossus, Dendrobates *sensu lato, *Epipedobates*, *Hyloxalu*s, and *Phyllobates* (Tables 1, 2).

### Pairwise correlations and logistic regressions

Our pairwise analyses (Table 3) show that the components of aposematism (i.e., conspicuousness and skin alkaloids) in dendrobatids are significantly and positively correlated (*P* = 0.030), but do not correlate with tadpole life history traits (*P* > 0.050 in all cases). These results suggest that aposematism does not covary directly with reproductive traits related to clutch size, number of offspring transported or tadpole body size after hatching. We did not find a significant correlation between tadpole size and egg size, but found a significant relationship between clutch size and the number of tadpoles carried by parents. Moreover, we found a positive relationship between alkaloid quantity and tadpole size. These results suggest that the aposematism and life history components form two clusters of traits that result in two separate emergent properties, namely anti-predator defenses (i.e., aposematism or crypsis) and reproductive strategies (i.e., offspring quality and parental care). However, these results were expanded and challenged when we incorporated them in our multivariate logistic regressions (see Table 4 and S3 for the complete model values).

**Table 3:** Comparison between ß values of independent phylogenetic regressions. The coefficient significance is indicated by *(superscript) *for P* < 0.05 and NS (superscript) for *P* > 0.05. All *P*-values were adjusted for false discovery rate (FDR). A longer version is provided in Table S4).

	Conspicuousness score	SSD	Clutch size	Egg size	Tadpole size	Tadpoles carried	Alkaloids
	–						
SSD	0.64^NS^	–					
Clutch size	− 0.001^NS^	0.003^NS^	–				
Egg size	0.028^NS^	− 0.015^NS^	− 2.53^NS^	–			
Tadpole Size	− 0.055^NS^	0.627^NS^	0.521^NS^	0.06^NS^	–		
Number of Tadpoles carried	− 0.012^NS^	0.005^NS^	0.578***	− 0.20^NS^	0.01^NS^	–	
Alkaloids	0.231*	0.003^NS^	0.001^NS^	0.215^NS^	− 0.05*	− 0.13^NS^	–

**Table 4:** Results of the phylogenetic logistic regression for parental care traits and aposematism components in poison frogs. Values in bold denote *P* < 0.05). The categorical variables and their the character states in parenthesis are: tadpole deposition site (phytotelmata/non-phytotelmata), cannibalism (presence/absence), alkaloids (presence/absence), tadpole-carrying sex (male/female), habitat where adults usually live (near streams/away from streams), conspicuousness (conspicuous/non-conspicuous), and type of care (male/female). The continuous variables include: Coloration score based on^43^, SSD (sexual size dimorphism), number of tadpoles transported, and size of tadpoles at Gosner stage 25. The asterisk (*) indicates that tadpole size was controlled by female body size.

Binary dependent	N	Intercept		Coloration score		SSD		Number of Tadpoles transported		Tadpole size*	
		β_0_	*P*	β_1_	*P*	β_2_	*P*	β_3_	*P*	β_4_	*P*
Tadpole deposition	112	− 255.207	0.087	− **224.170**	**0.001**	348.390	0.532	**125.920**	** < 3e−04**	− 1.122	0.953
Cannibalism	23	− 7.620	0.769	573.730	0.614	− 952.465	0.366	**− 224.700**	**0.038**	− 19.480	0.327
Alkaloids	61	**−** **693.700**	**0.003**	**497.460**	** < 3e**−**04**	856.480	0.324	− **136.360**	**0.013**	− 15.210	0.383
Tadpole−ferrying sex	110	− 47.990	0.800	114.230	0.198	847.570	0.081	− 0.350	0.988	11.302	0.179
Conspicuousness	114	− 227.266	0.084	−	−	697.208	0.242	21.313	0.650	6.207	0.403
Habitat	114	**226.180**	**0.045**	**255.790**	**0.014**	− 851.212	0.064	− **90.205**	**0.0134**	− 11.880	0.100
Type care	82	194.769	0.402	− 6.936	0.949	700.860	0.286	1.352	0.985	9.757	0.451

Tadpole deposition in phytotelmata tends to occur more often in conspicuous species which, in turn, carry fewer tadpoles at once (Table 4, *P* < 0.003 in both cases). In contrast, species that transport tadpoles to non-phytotelmata water bodies tend to be cryptic and carry a much larger number of tadpoles. We found no differences in tadpole size (controlled by female body size) between phytotelm-breeders and non-phytotelm-breeders (*P* = 0.840). Because species that exhibit egg-feeding have very small eggs and tadpoles with a small amount of yolk reserves (hence the need of food supplementation by the mothers) (Summers & McKeon 2004), they can potentially bias these results. For this reason, we also ran the phylogenetic logistic regression excluding egg-feeding species. However, as shown in Table S5, the results did not change.

Species with territories away from streams tend to have more conspicuous coloration and transport fewer tadpoles than those that live in riparian sites (*P* ~ 0.013). We did not find differences in SSD or tadpole size when comparing between tadpole deposition sites (*P* > 0.050). Likewise, no associations were found between SSD and clutch size or tadpole length (*P* > 0.050 in all cases).There were no differences in the number of tadpoles transported, SSD, tadpole size, and coloration between species with male-biased and female-biased care (*P* > 0.050). Species that exhibit larval cannibalism tend to carry fewer tadpoles than species without cannibalism (*P* ~ 0.038), yet no differences in SSD, tadpole body size or coloration were detected in association with cannibalism (*P* > 0.050). Finally, when only the presence of alkaloids was accounted for in our analyses, species with skin alkaloids tend to carry fewer tadpoles at once than species without alkaloids (*P* ~ 0.003).

We also tested whether the components of aposematism (conspicuousness and skin alkaloids) affect the transition of tadpole deposition site from phytotelmata to non-phytotelmata and vice versa. For this purpose, we tested if a pair of binary traits for aposematism components and tadpole disposition site have evolved independently (Pagel 1994; Pagel and Meade 2006) using the BayesTraits framework (Barker et al. 2007). This approach allowed us to compare the fit between a model that considers the traits to have evolved independently and models which assume that the traits’ evolution depends on one another (i.e., present correlated evolution); these models can be modified to present different constraints on their transition rate parameters.

**Figure 3﻿:**
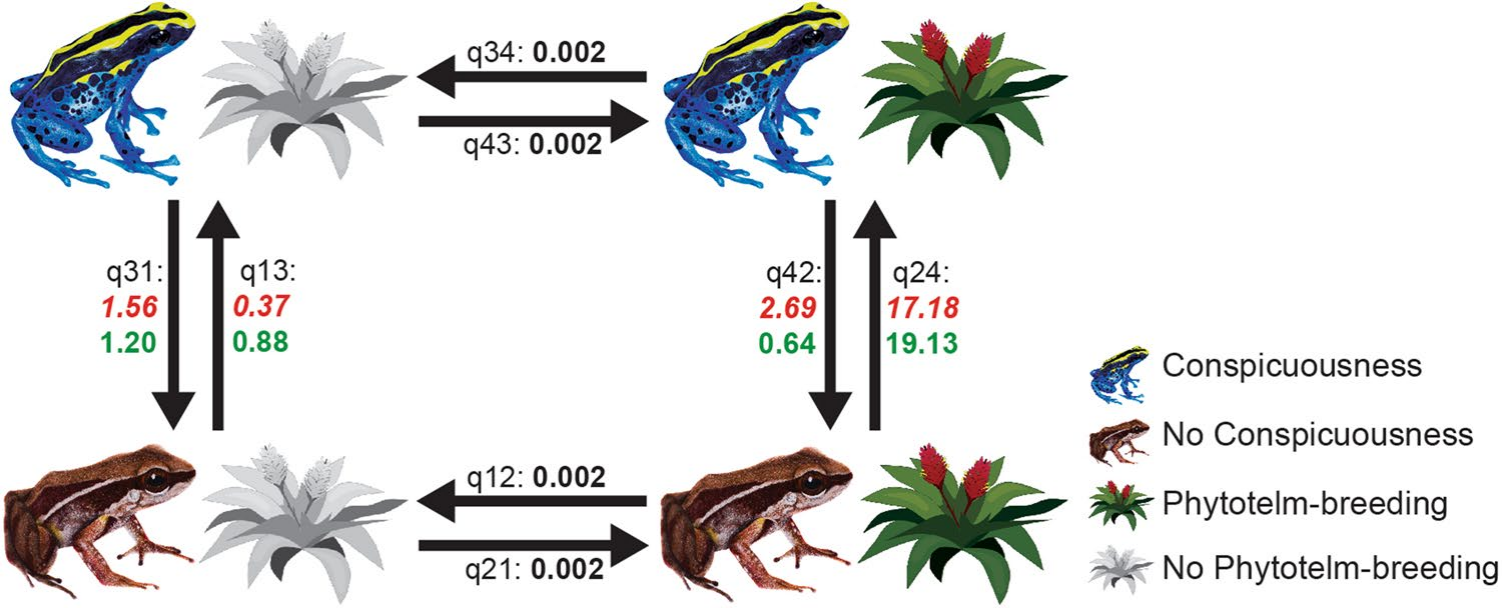
Transition rate estimates from the best dependent model with *q34* = *q43* = *q12* = *q21* constraints for both conspicuousness and skin-alkaloids with phytotelm-breeding derived from MCMC analyses. Values in red and italics correspond to the model with conspicuousness traits and values in green (no italics) correspond to the model with skin-alkaloids traits. Note that transition values at *q34, q43, q12*, and *q21* have the same transition value in both types of models. Illustrations by MJ Tovar-Gil, AM Ospina-L and DL Rivera-Robles. Figure was modified using Adobe Illustrator version 15.1.0 (https://www.adobe.com/). The image of the phytotelm is under Non-Commercial Use (http://clipart-library.com).

Our results provide strong evidence of dependent evolution between aposematism components and phytotelm-breeding (Bayes factor-BF = 39.314; see Fig. 3, Tables 4 and S6–S8 for details in all values and comparation between models). Briefly, when comparing the independent and dependent models, we found the latter to be the best fit (i.e., for both conspicuousness and skin alkaloids with phytotelm-breeding). For the test between conspicuousness and tadpole deposition site, we found that the highest transition rate estimate was from non-conspicuous to conspicuous when phytotelm-breeding is present (*q24* = 17.18). When skin alkaloids are incorporated in the model instead of conspicuousness, the highest transition rate was from absence to presence of alkaloids when phytotelm-breeding is present (*q24* = 19.13). The reversal rates in these models, i.e., when conspicuousness and alkaloid presence favor the transition to phytotelm-breeding, were much smaller (i.e., *q31* = 1.56 for conspicuousness and *q31* = 1.20 under alkaloid presence). Overall, phytotelm-breeding species have a rate of transition to aposematic phenotype (e.g., warning coloration and alkaloid defenses) that is 17–19 times the rate of the opposite transition, i.e., reversal to the ancestral cryptic and non-defended phenotypes. Table 4 and Fig. 3 provide details on transition rate estimates of all character state combinations and all models tested.

## Discussion

One of the costs of parental care is the increased predation risk or the caregivers through prolonged exposure to predators while provisioning and protecting their young. These costs can be partially mitigated if the parents exhibit warning colors that signal predators about the presence of defenses such as toxic or noxious compounds (e.g., alkaloids, venoms, etc.). For example, adult burying beetles (*Nicrophorus vespilloides*), which are known for their elaborate parental care, exhibit a conspicuous warning coloration coupled with an anal exudate that deters potential predators^87^. Using a phylogenetic framework, we investigated whether aspects of poison frog ecology and life history covary with their parental care behavior. Our findings indicate that, indeed, aposematism may have facilitated the diversification of parental care strategies in dendrobatids. Therefore, we propose that aposematism might serve as an “evolutionary platform” where parental behavior can further diversify as predation pressure is reduced.

Results of the ancestral reconstruction indicate that aposematism (i.e., conspicuousness and alkaloid sequestration) evolved first than most complex reproductive behaviors in dendrobatids. For instance, our tests of correlation between aposematism and tadpole deposition site (BayesTraits analyses) suggest that, once this defensive strategy is established, phytotelm-breeding behavior requires aposematism for its maintenance. Furthermore, dendrobatids that use phytotelmata for tadpole depositionmay become more colorful and more toxic than non-phytotelm-breeding species. In fact, phytotelm-breeding species have transition rates from cryptic to aposematic coloration that are about 17 times the change rates in the opposite direction (Fig. 3, 2.69 times). Likewise, the transition rate in species with phytotelm-breeding suggests that alkaloid sequestration is favored almost 19 times more than in the opposite direction (Fig. 3, 0.6 times). This suggests that if phytotelm-breeding is lost, most species that already have conspicuous coloration or sequester alkaloids are less likely to lose both traits.

We suggest several evolutionary scenarios for the association between aposematism and parental care. First, our ancestral reconstructions suggest that aposematism appeared before phytotelm-breeding and other complex reproductive behaviors, but once these complex behaviors are established, losing aposematism is less likely. Second, aposematism also seems to further the diversification of parental care strategies (e.g., biparental care) due to reduced predation pressure. Third, the low rates of reversal from the aposematic to the non-aposematic phenotype in phytotelm-breeding species suggest a higher predation pressure in species that lack or lost the aposematic phenotype, which might result in increased extinction rates. Overall, aposematism is necessary to maintain complex reproductive behaviors, like phytotelm-breeding, but this trait can serve for further diversification of other parental care strategies (e.g., egg provisioning).

Field evidence supports our results on the correlated evolution of aposematism and phytotelm-breeding, which might have enabled the further diversification of microhabitat use in the context of reproduction in aposematic dendrobatids. In species that transport tadpoles to arboreal phytotelmata, parents could be under high predation risk as they climb trees without concealing structures (e.g., leaf litter) and with reduced understory coverage, limiting the availability of places to hide if detected by a potential predator^25,88,89^. Parents may also prefer to go far from their territories to deposit their tadpoles as an offspring dispersal strategy to reduce competition and inbreeding^33,90^. Given the efficacy of aposematism in poison frogs, long forays while caring for offspring are likely safer for aposematic than for cryptic species. Aposematism might not only warn predators about unprofitability, but it may also allow defended frogs caring for their offspring to widen their core territories. These areas tend to be larger in aposematic than in cryptic dendrobatids^90^, thus allowing aposematic species enhanced access to food resources and shelter^91^.

The evolutionary transition of tadpole rearing sites from non-phytotelmata waterbodies to phytotelmata is assumed to be a strategy to reduce or avoid large predators, which are abundant in ponds and streams^47,92–94^. However, phytotelmata are harsh environments with low-nutrient availability and a high desiccation risk. As a consequence, tadpoles deposited in such sites may require further investment by parents to be able to complete their development to metamorphosis^29,47,55^. Thus, it is not surprising that species whose tadpoles develop in phytotelmata had evolved food provisioning with unfertilized eggs. This harsh environment with limited resources and an ephemeral nature seems to facilitate the evolution of larval cannibalism as a facultative behavior and, in some instances as an optimal foraging strategy.

The shift from using large waterbodies to phytotelmata may have been accompanied by adaptations such as the observed reduction in clutch size and, consequently, in the number of tadpoles transported (Fig. 1 and Table S1). We propose two possible, non-mutually exclusive explanations for this change in aposematic species. First, because tadpoles occupy a significant area on the back of the parents, a high number of tadpoles on the parent’s back might interfere with the display of their warning signal, thus increasing predation risk. In other words, fewer tadpoles on the parent’s back may prevent the concealment of the warning signal during tadpole transport. Some species of aposematic frogs transport a large number of tadpoles on their back (e.g., *Ameerega trivittata*). In this case, we hypothesize that the conspicuous head coloration of the parent could be enough to warn a potential predator about its unprofitability. Alternatively, the potentially disruptive pattern created by tadpoles on the back of the parent could confuse would-be (and maybe inexperienced) predators. Both ideas could be tested experimentally, for example, either using clay models of aposematic frogs with markings that mimic tadpoles on their back and determining if these models elicit more attacks by predators than tadpole-free models, or testing predation risk upon prey (e.g., mealworms) placed on printed drawings of poison frogs, and using naïve chickens as predators^95,96^.

An alternative explanation for the clutch size reduction is that natural selection can promote a small brood size under Lack's principle^97,98^. According to this hypothesis, dendrobatid frogs with restrictive tadpole deposition sites may have evolved an optimal clutch size that corresponds with the largest number of tadpoles that the parents can care for^47,119^. Likewise, biparental care should have evolved along with egg provisioning as a direct food resource that only females can provide by sacrificing large gametes. These tactics would thus be part of an optimal life history strategy to deal with the limited resources and space in phytotelmata. Variants of this hypothesis have been proposed for some *Dendrobates* species (e.g., *D. variabilis*, *D. imitator*) that use arboreal phytotelmata for tadpole development^55,99–101^. We consider that our hypothesis could also explain the small clutch size for other non-aposematic genera such *Anomaloglossus* that also use phytotelmata as deposition sites^30,102^.

A prediction derived from Lack's principle is that parents must invest time to test the quality of phytotelmata, and they should have evolved high cognitive abilities to memorize the spatial location of the clutch to minimize travel time for food provisioning (see also^103,104^). Therefore, particularly in species without biparental care, unfertilized egg provisioning may require the evolution of a high cognitive capacity in the mothers to remember where their offspring are located in tridimensional space^120–122^. If the provisioning of females to their offspring is absent or inefficient, this might have enabled the facultative occurrence of tadpole cannibalism. In this case, if more than one individual is deposited in a given pool, cannibalism might allow tadpoles to prey on other individuals as an optimal strategy to avoid starvation^103^. This inference implies that most species using phytotelmata as rearing sites should exhibit tadpole cannibalism, which appears not to be the case. Larval cannibalism has been studied in only a few species^37,95,105–109^ and, in some instances, it appears to be facultative or opportunistic. The fact that few instances of cannibalistic behavior have been documented might be the result of limited observations, and we thus propose that this behavior might be more widespread than currently thought.

From the male’s perspective, tadpole transport can be especially costly in terms of territorial ownership^110^ and in time invested for calling to attract additional mates^111^. This is because dendrobatid males are often polygamous and can take care of multiple egg clutches, laid by different females^60,90,112^. In species that use phytotelmata, males might be selected to invest less than females in finding optimal phytotelmata with specific features for tadpole deposition, for example, without non-kin tadpoles^93^. Under this ecological scenario, natural selection could have favored higher cognitive capacities in females than males. Likewise, it should have favored tadpole transport to high quality phytotelmata based on multiple parameters such as food availability, number of potential depredators,and physicochemical characteristics of the water, and egg provisioning if nutrients are deficient^120–122^. However, it remains to be determined if this hypothesis applies to non-aposematic species such as the three species of *Colostethus* in which females transport their tadpoles to streams and larval cannibalism is absent^57^. One potential explanation for the latter case is that males tend to decrease their care of an older clutch in a set of clutches with multiple females, and mothers of this older clutch tend to transport their tadpoles in cases in which males do not do so^113^. All of these possible explanations warrant further investigation, ideally with an experimental approach.

Previous studies suggest that territories near streams might be the ancestral habitat of dendrobatids and their hyloid relatives^47^. However, our results suggest that the ancestral habitat of poison frogs was away from streams. This alternative hypothesis finds support in other phylogenetic hypotheses stating that the sister group of Dendrobatidae could be Bufonidae (Pyron and Wiens 2011^114^), Leptodactylidae^115^ or Craugastoridae/Eleutherodactylidae^117^. All of which have numerous species occurring in terrestrial habitats. Under this scenario, we infer that the ancestral condition for Dendrobatidae is the terrestrial habitat, whereas the riparian habitat seems to be derived. In support of this inference, similar results on habitat evolution across anurans were found by Moen et al. (^118^; see this paper’s supplementary materials). In their study, the ancestral microhabitat of Dendrobatidae was suggested to be terrestrial and away from streams. For amphibians, terrestrial habitats are hostile environments due to the increased risk of desiccation, unless a parent provides much needed moisture (e.g., watery urine) to developing offspring in terrestrial sites, but also across distant taxa such as insects (e.g., thrips^116^).

Studying the reproductive strategies and parental care in poison frogs is fundamental to understanding diversification patterns within the Dendrobatidae^55,99–101^. While a terrestrial environment appears to be ancestral habitat of poison frogs and is common to other hyloid clades (e.g., Leptodactylidae and Bufonidae), the evolution of more complex tadpole deposition sites such as phytotelmata likely imposed a strong selection pressure in early dendrobatids. With the evolution of aposematism and the concomitant reduction in predation risk, complex behaviors might have emerged and further diversified into the remarkable spatial/cognitive abilities, elaborate biparental care, and nutritious egg provisioning seen today in some dendrobatids. Therefore, while we do not claim that aposematism caused complex parental behaviors to evolve in the first place, we suggest that aposematism might serve as an umbrella trait against predation. Once phytotelm-breeding appears in aposematic species, this defensive strategy is less likely to revert to the non-aposematic phenotype. Likewise, aposematism and phytotelm-breeding seem to have allowed further diversification of parental care behavior. Phytotelm-breeding, which may have originated as a response to the high predation pressure in streams, requires the evolution of further strategies for tadpoles to withstand, and thrive in, sites with low nutrient availability. Consequently, a small clutch size, egg provisioning, and cannibalism may be further adaptations in species with tadpoles that develop in phytotelmata which, in turn, may require higher parental investment and may have favored the evolution of complex offspring-caring activities, specially by female parents, towards their offspring.

## Methods

Dendrobatids include > 310 species with widespread distribution (e.g., from lowlands to paramos in the Andes) across Central and Northern South America^43,66,67^. We obtained morphological and behavioral traits associated with parental care in 289 species of dendrobatids (aposematic: 91 species and cryptic: 198 species). Information about those traits was obtained from the scientific literature, examination of vouchers in biological collections (for morphological information), and personal observations in the field (Tables 1 and 2). Detailed summary datasets and references are provided in Tables S1–S2. We provide a brief description of the data used in our analyses below.

### Parental care and tadpole life history traits (discrete)

Our analyses include traits measured both as continuous and discrete binary variables. In the case of multistate discrete traits, we binarized such phenotypes using their most common or their broadest definition, as required for phylogenetic logistic regressions. These traits included the sex of the caregiver (usually characterized as none, male, female or both), sex of the transporting parent, and tadpole deposition sites (usually classified as streams, ponds, and phytotelmata). In the case of sex of the caregiver (113 species with reports, Tables 1, 2), we binarized all known cases as male-biased or female-biased, excluding from analyses those which could not be unambiguously binarized (see below). The male-biased state (92 species, Tables 1, 2) was assigned when most of the care (i.e., clutch attendance and tadpole transport) was done by the male, while the female only contributed sporadic care or gametes. The female-biased state (10 species, Tables 1, 2) was assigned when females contributed more to the parental care than males by providing periodic unfertilized eggs to developing tadpoles. We excluded species with no apparent parental care (i.e., 3 species or 2.65% of the diversity of species with reports) or in which we could not assign unambiguously a dominant sex involved in parental care (i.e., 8 species or 7.07% of species with reports). In both of these excluded groups, we assumed that the available reports reflected limited observations and thus required further investigation in the field.

We assigned of the sex of the tadpole-carrying parent based on the sex that is most frequently observed. It is not uncommon in some species that both males and females transport tadpoles (Tables S1–S2), yet the frequency of such events is usually much higher in one sex. For example, both parents of *Hyloxalus elachyhistus* may transport tadpoles, but the ratio is five to one male to female as the ferrying parent^68^. Thus, in this (and similar) cases, we assigned the sex of tadpole-carrying parent as male. We also designated the habitat of the parents on the basis of their typical habitat (near-riparian) or away from streams (terrestrial-forest floor). This qualitative trait represents the habitat where each species lives during most of its life cycle, as many taxa are limited to streams while others spend most of their time on the forest floor away from streams, although they may range more widely in the habitats and sites where they deposit tadpoles.

We binarized tadpole deposition sites as non-phytotelmata and phytotelmata, considering the latter as the most specialized tadpole deposition site; species which could use both ponds and streams for tadpole deposition were assigned to the ‘non-phytotelmata’ category. Most dendrobatids deposit their tadpoles in non-phytotelmata environments which are rich in nutrients (e.g., algae and detritus), and their larvae are herbivorous. In contrast, phytotelmata, water-holding structures formed in terrestrial or epiphyte plants (e.g., modified leaves, leaf axils, and tree holes), are characterized by low nutrients (e.g., lack algal debris). Therefore, tadpoles developing therein are usually entirely dependent on the food provisioned by their mother or have evolved facultative cannibalism to acquire basic nutrients if parental investment is limited only to transport. We thus scored larval cannibalistic behavior as present or absent.

In sum, the binary (qualitative) variables associated with parental care and tadpole life history were: (1) sex of the main caregiver (male or female), (2) sex of tadpole carrier (male or female), (3) habitat of the parents (near or far from streams), (4) tadpole deposition site (non-phytotelmata or phytotelmata), and (5) tadpole cannibalism (present or absent).

### Parental care and tadpole life history traits (continuous)

For quantitative traits we included sexual size dimorphism (SSD), which was calculated as the ratio between female’s and male’s body length measured in mm. SSD is a common measurement of the intensity of sexual selection acting on male and female body size, and could have an effect on behavior and reproductive traits due to an allometric relationship^69–71^. For example, females with larger body size could have more eggs per clutch, males with smaller body size could be able to transport fewer or smaller tadpoles. We also included the following continuous variables, which are associated with parental investment: mean clutch size, defined as the number of eggs per clutch; mean egg size, measured as egg diameter in mm; mean number of tadpoles simultaneously transported by a parent; and body length of transported tadpoles (measured in mm on tadpoles at the 25 developmental stage sensu^72^, which is the larval stage at which tadpoles are transported). We controlled the egg size, clutch size and tadpole size by female size, and we used these residual values in posterior analysis. For all these variables, we used the median of available ranges when only these were reported in the literature. All variables (i.e., not residuals values) were log-transformed to improve their distribution when used in statistical analyses.

### Aposematism-related traits

To account for warning signal, we assigned a relative index of conspicuousness to each species based on a quantification of color description made by multiple independent human observers (sensu^43^). While we are aware that human vision is different from that in other animals^73,74^, and that there are established methods to reliably measure animal color patterns and assess how receivers (e.g., predators and conspecifics) see them^74–78^, we opted for this method for the following two reasons. First, previous research indicates that humans are able to detect a considerable portion of color variation in the visible range^79^. Second, this approach allowed us to cover the entire 220 taxa (i.e., 68% of the known >310 dendrobatid species) that were included in our analyses where such information could be derived from color descriptions of live male specimens were derived from species descriptions, photographs, field notes by independent observers (multiple independent human observers; mean value of 2.8 ± 1.17; range 1–6 observations). Although measuring reflectance spectra or taking standardized photographs for further analyses would be ideal, for this number of species it would be logistically nearly impossible, as many of these species are distributed across the Neotropics, there are not recent reports of their conservation status, and some are extremely hard to find.

Coloration scores were taken from^43^. The frog's body was divided into 11 non-overlapping skin segments covering dorsal, ventral, and lateral views, and the presence-absence of dorsal stripe(s), lateral stripe(s), and flash marking in arms and legs. Based in human vision perception of chromatic contrast each skin segment of the body was scored with 1 (i.e. conspicuous; red, yellow, blue, orange, green) if it contrasts against a natural background [leaf litter (i.e., gray, brown, and black)] or 0 (non-conspicuous) if otherwise. For each species, an overall coloration score was derived from the sum of all the individual segment scores, which provided a continuous estimate of coloration, ranging from 0 (all Sects. 0 or 0 × 11 = 0; no contrast) to 11 (all Sects. 1 or 1 × 11 = 11; maximum contrast). Additionally, we binarized this coloration score, such that taxa with coloration scores of ≥ 6 were considered as conspicuous, whereas taxa with coloration scores of < 6 was considered as non-conspicuous. This 6-points threshold is based on the observation that all species reported to have skin alkaloids (i.e., chemical defenses) have scored at least 6-points and all these species have color usually considered aposematic (e.g., blue, yellow, red on their dorsum). For further details, and possible constraints and limitations of this methodology, see^43^. To help the readers get a better idea of how we calculated the color scores, see to illustrate the skin segments of each frog in ^43^. We have also included a table in the supplementary materials with the raw coloration scores (Table S5). To account for the presence of chemical defense, we also classified each species based on the presence (> 10 µg of alkaloids per 100 mg skin) or absence (< 10 µg of alkaloids per 100 mg skin) of skin alkaloids, as reported in the literature (Tables S1–S2). In sum, we included as continuous variables: (1) conspicuousness index (a value between 0 and 11), and (2) alkaloid measurement score as a proxy of μg of alkaloids per 100 mg of skin; and as qualitative variables: (1) coloration (conspicuous or non-conspicuous) based on a 6-points threshold as indicated above, and (2) chemical defense (present or absent) based on whether or not the species has been reported to have skin alkaloids.

### Statistical analyses

For the phylogenetic comparative analyses, we used the time-calibrated phylogeny of Dendrobatidae as provided by^43^ and updated based on^80^ With our data matrix and this phylogeny, we estimated the likelihood of marginal ancestral state reconstruction^81^ of conspicuousness, sex of the caregiver, habitat, sex of tadpole-carrying parent, tadpole deposition site and occurrence of cannibalism in tadpoles. For this, we used an all-equal rates like (ER) parameter of transition among traits using the R-package ‘ape’ version 5.3^82^. To test for the relationship between continuous traits and parental care, we performed a pairwise comparison between values resulting from the independent phylogenetic regressions; we adjusted *P*-values for False Discovery Rates (FDR^83^). Then, to explore whether continuous variables (log transformed) explained conspicuousness, sex of the caregiver, habitat, sex of tadpole-carrying parent, tadpole deposition site and tadpole cannibalism, we performed phylogenetic multivariate logistic regressions using the R-package ‘MCMCglmm’ version 2.29^84^. For these analyses, we used the dendrobatid chronogram to derive the inverse of the matrix of phylogenetic correlations and a list of priors, which includes the default prior for the fixed effects and a set of priors for the random effects (G), and residual variance (R) that correspond to an inverse-Gamma distribution with shape and scale parameters equal to 0.001. The number of iterations was set to 20 × 10^6^ with a burn-in parameter of 1000 and a thining parameter of 500. We ran a total of four Monte Carlo Markov Chains with four independent starting points and the convergence was checked after all runs. We used the Gelman–Rubin convergence diagnostic^85^ to determine the convergence of runs. This diagnostic evaluates MCMC convergence by analyzing the difference between multiple Markov chains. The convergence is assessed by comparing the estimated between-chains and within-chain variances for each model parameter. Values close to 1 indicate convergence in the models. The values are reported in Table S3 in the Supplementary materials.

Finally, we analyzed whether coloration (or presence of alkaloids) and breeding site have evolved independently or not using BayesTraits v3.0.2^86^ For this purpose, we applied the discrete function to our data and estimated the fit of independent and dependent models under a Bayesian framework. First, we tested whether the coloration (or absence or presence of alkaloids) and breeding site evolved independently (independent model). Second, we tested whether the rate of change in one trait exhibits an evolutionary dependence on the value of the other trait (dependent model). We estimated the marginal likelihood using a stepping-stone sampler with 100 stones and 10,000 iterations per stone. We employed the marginal likelihood of complex and simple models to obtain the Log Bayes Factor (logBF) to test whether the traits are evolutionary correlated between the independent and the dependent model. Bayes Factor values were considered as weak evidence of evolutionary correlation if LogBF < 2; positive evidence of evolutionary correlation if LogBF > 2; strong evidence if LogBF was 5–10; and very strong evidence if LogBF > 10. We further modified the dependent models to improve its fit by constraining the transition rates *q34* = *q43* = *q12* = *q21* (Tables 5 and S6–S8). Finally, we examined the potential evolutionary pathways of the traits using the posterior distributions of the eight transitions rates between four possible combination of character states (i.e. coloration and phytotelm-breeding, coloration and no phytotelm-breeding, no coloration and phytotelm-breeding, no coloration and no phytotelm-breeding). We repeated the same procedure as described above but including presence of alkaloids in our models (i.e. alkaloids and phytotelm-breeding, alkaloids and no phytotelm-breeding, no alkaloids and phytotelm-breeding, no alkaloids and no phytotelm-breeding).

**Table 5:** Parameters of the dependent models using aposematism components (conspicuousness or skin-alkaloids) versus phytotelm-breeding traits.

Dependent on	Trait	Parameter	Transitions
Conspicuousness = 1	Phytotelm-breeding	q34	0 → 1
or Skin-Alkaloids = 1		q43	1 → 0
Conspicuousness = 0	Phytotelm-breeding	q12	0 → 1
or Skin-Alkaloids = 0		q21	1 → 0
Phytotelm-breeding = 1	Conspicuousness	q24	0 → 1
	or Skin-Alkaloids	q42	1 → 0
Phytotelm-breeding = 0	Conspicuousness	q13	0 → 1
	or Skin-Alkaloids	q31	1 → 0

## Ethics declarations

Information was obtained from literature, examination of vouchers in biological collections (for morphological information), and personal observations in the field. All methods were carried out in accordance with relevant guidelines and regulations. No experimental protocols were performed.

## Supplementary Information

Supplementary Information 1.

Supplementary Information 2.

## Data Availability

The datasets supporting this article will be uploaded to the online repository figshare (https://figshare.com/s/338fc287ef792d432bb8).
