# Supplementary material for: Aposematism facilitates the diversification of parental care strategies in poison frogs
Source: Sci Rep. 2021 Sep 24;11:19047. doi: 10.1038/s41598-021-97206-6 (PMC8463664; doi:10.1038/s41598-021-97206-6)
Supplement: Supplementary file 2 — Supplementary Information 2. [file 41598_2021_97206_MOESM2_ESM.docx]

Table S6. Test results based on log Bayes Factors (*log* BF) between independent and dependent models based on conspicuousness and phytotelmata-breeding from 160 taxa.

| Model  type | Dependent on | N rates | Rates constraints | Log marginal likelihood | *log*BF  (vs Independent) | *log*BF  (vs full model) | *log*BF  (vs best model) |
| --- | --- | --- | --- | --- | --- | --- | --- |
| Independent | None | 4 | both-independent | -117.377 | -- | 2.072* | 39.314** |
|  |  | 5 | *q34 = q43 = q12 = q21* | -97.720 | 39.314** | -37.243 | -- |
| Dependent | Conspicuousness | 6 | *q34 = q43; q12 = q21* | -105.248 | 24.257** | -22.186 | -15.057 |
|  |  | 6 | *q34 = q12; q43 = q21* | -105.746 | 23.262** | -21.191 | -16.052 |
|  |  | 5 | *q24 = q42 = q13 = q31* | -122.000 | -9.245 | 11.317** | -48.560 |
| Dependent | Phytotelmata-breeding | 6 | *q24 = q42; q13 = q31* | -129.170 | -23.586 | 25.657** | -62.900 |
|  |  | 6 | *q24 = q13; q42 = q31* | -123.570 | -12.385 | 14.457** | -51.700 |
| Dependent | Full model | 8 | all-free | -116.341 | 2.072* | -- | -37.243 |

*log*BF: if <2 weak evidence; if >2 and * complex model is favored; if >10 and ** complex model is strongly favored

Table S7. Test results based on log Bayes Factors (*log* BF) between independent and dependent models based on skin-alkaloids and phytotelmata-breeding from 71 taxa.

| Model  type | Dependent on | N rates | Rates constraints | Log marginal likelihood | *log*BF  (vs Independent) | *log*BF  (vs full model) | *log*BF  (vs best model) |
| --- | --- | --- | --- | --- | --- | --- | --- |
| Independent | None | 4 | both-independent | -52.828 | -- | -12.163 | 17.025** |
|  |  | 5 | *q34 = q43 = q12 = q21* | -44.316 | 17.025** | -29.189 | -- |
| Dependent | Skin-Alkaloids | 6 | *q34 = q43; q12 = q21* | -50.994 | 3.669* | -15.832 | -13.356 |
|  |  | 6 | *q34 = q12; q43 = q21* | -51.042 | 3.572* | -15.736 | -13.453 |
|  |  | 5 | *q24 = q42 = q13 = q31* | -47.407 | 10.843** | -23.006 | -6.183 |
| Dependent | Phytotelmata-breeding | 6 | *q24 = q42; q13 = q31* | -53.027 | -0.398 | -11.765 | -17.424 |
|  |  | 6 | *q24 = q13; q42 = q31* | -51.945 | 1.767 | -13.931 | -15.258 |
| Dependent | Full model | 8 | all-free | -58.910 | -12.163 | -- | -29.189 |

*log*BF: if <2 weak evidence; if >2 and * complex model is favored; if >10 and ** complex model is strongly favored

Table S8. Transition rate estimates from the best dependent model with *q34 = q43 = q12 = q21* constraints for both conspicuousness and skin-alkaloids with phytotelmata-breeding derived from MCMC analyses. Values under parameter name is its mean and 95CI. For description of each parameter see Table 4 and.

|  |  | Phytotelmata-breeding | | | |
| --- | --- | --- | --- | --- | --- |
|  |  | 0,0 | 0,1 | 1,0 | 1,1 |
| Conspicuousness | 0,0 | -- | ***q12***  0.0024  (0.0023 – 0.0024) | ***q13***  0.3779  (0.3373 – 0.4185) | 0 |
|  | 0,1 | ***q21***  0.0024  (0.0023 – 0.0024) | -- | 0 | ***q24***  17.1817  (16.8461 – 17.5173) |
|  | 1,0 | ***q31***  1.5607  (1.4029 – 1.7185) | 0 | -- | ***q34***  0.0024  (0.0023 – 0.0024) |
|  | 1,1 | 0 | ***q42***  2.6913  (2.6293 – 2.7534) | ***q43***  0.0024  (0.0023 – 0.0024) | -- |
|  |  |  | | | |
|  |  | 0,0 | 0,1 | 1,0 | 1,1 |
| Skin-Alkaloids | 0,0 | -- | ***q12***  0.0025  (0.0024 – 0.0025) | ***q13***  0.8826  (0.7951 – 0.9702) | 0 |
|  | 0,1 | ***q21***  0.0025  (0.0024 – 0.0025) | -- | 0 | ***q24***  19.1342  (18.7726 – 19.4958) |
|  | 1,0 | ***q31***  1.2062  (1.0863 – 1.3262) | 0 | -- | ***q34***  0.0025  (0.0024 – 0.0025) |
|  | 1,1 | 0 | ***q42***  0.6478  (0.6233 – 0.6723) | ***q43***  0.0025  (0.0024 – 0.0025) | -- |
